# Synthesis and toxicity assessment of *Coffea arabica* extract-derived gold nanoparticles loaded with doxorubicin in lung cancer cell cultures

**DOI:** 10.3389/fbioe.2024.1378601

**Published:** 2024-04-26

**Authors:** Isaí Trejo-Teniente, Blanca Estela Jaramillo-Loranca, Genaro Vargas-Hernández, Maricela Villanueva-Ibáñez, Xochitl Tovar-Jiménez, Patricia Nayeli Olvera-Venegas, José Tapia-Ramírez

**Affiliations:** ^1^ Laboratory of Nanotechnology, New Materials and Systems for Health, Industry and Alternative Energies, Universidad Politécnica de Pachuca, Zempoala, Hidalgo, Mexico; ^2^ Laboratory of Bioactive Compounds, Universidad Politécnica de Pachuca, Zempoala, Hidalgo, Mexico; ^3^ Biotechnology Engineering Education Program, Universidad Politécnica de Pachuca, Zempoala, Hidalgo, Mexico; ^4^ Department of Genetics and Molecular Biology, Centro de Investigaciones y de Estudios Avanzados IPN, Mexico City, Mexico

**Keywords:** gold nanoparticles, biosynthesis, *Coffea arabica extract*, nanohybrids, doxorubicin, ligands, cancer

## Abstract

Cancer is the second leading cause of death worldwide, despite the many treatments available, cancer patients face side effects that reduce their quality of life. Therefore, there is a need to develop novel strategies to increase the efficacy of treatments. In this study, gold nanoparticles obtained by green synthesis with *Coffea arabica* green bean extract were loaded with Doxorubicin, (a highly effective but non-specific drug) by direct interaction and using commercial organic ligands that allow colloidal dispersion at physiological and tumor pH. Conjugation of these components resulted in stable nanohybrids at physiological pH and a tumor pH release dependent, with a particle size less than 40 nm despite having the ligands and Doxorubicin loaded on their surface, which gave them greater specificity and cytotoxicity in H69 tumor cells.

## 1 Introduction

Cancer encompasses a broad group of diseases characterised by rapid proliferation and spread of abnormal cells, potentially impacting multiple organ systems within the organism. Unlike healthy cells, new abnormal cells reproduce even when they are not required and move to distant areas of the body through the circulatory and lymphatic systems by metastasis (American Cancer Society, 2015) (WHO, 2021). Currently, cancer is the second leading cause of death in the world, with one in every six deaths being cancer related. In 2020, cancer caused 10 million deaths. The main types of cancer worldwide are: 1) lung with 1.8 million deaths; 2) colorectal with 935,000 deaths; 3) liver with 830,000 deaths; 4) gastric with 769,000 deaths and 5) breast with 685,000 deaths (WHO, 2021).

Despite multiple existing therapies and medical advances, chemotherapy remains the most effective clinical treatment, although it remains associated with nonselective toxicity and severe side effects (Mioc et al., 2019). Being self-cells, it is difficult to treat cancer cells without harming healthy tissues because chemotherapy also destroys and slows down the proliferation of healthy cells (NIH, 2015). Therefore, there is an urgent need for more effective alternative treatments. Nanotechnology has emerged to diagnose, monitor, and treat this condition (Connor and Broom, 2018). Nanoparticles target cells owing to their tumour-specific characteristics, such as increased permeability and vulnerability (Dhamecha et al., 2016). Likewise, nanoparticles are used in Trojan horses to transport cytotoxic drugs, antibiotics, and immunomodulators (Thambiraj et al., 2019; Yeganeh et al., 2022). Among the materials frequently used as drug carriers such as biopolymers (Garg et al., 2022), inorganic or metallic (Jeevanandam et al., 2022; Garg et al., 2024¸ Krishnan et al., 2021), gold nanoparticles (AuNPs) have great potential for cancer treatment. On the one hand, this is owing to the rapid accumulation of AuNPs in cancer tissue facilitated by the enhanced permeability and retention of immature blood vessels supplying the cancer tissue (Maheda, 2021; Fatrekar et al., 2021). Additionally, their ability to traverse biological membranes is noteworthy. Gold nanoparticles ranging from 10 to 100 nm are endocytosed by cells and are observed in clathrin- and caveolin-coated vesicles (Darweesh, et al., 2019), specifically in lung cancer cells (Woodman et al., 2021). Physical, chemical, and biological methods are employed to obtain nanoparticles, with the latter being cost-effective and eco-friendly (Jeevanandam et al., 2022). In addition, the green synthesis of AuNPs offers the advantage of functionalizing their surface with biomolecules from plant extracts, such as flavonoids, alkaloids, phenols, terpenes, polyphenols, alcohols, sugars, and proteins, which act as reducing agents to produce them (Siddiqi and Husen, 2017). *Coffea arabica* is among the most attractive plants for AuNP biosynthesis since it contains a wide range of phenolic compounds and their derivatives (El-Nabi et al., 2018) that possess health benefits (Kolb et al., 2020). It has recently attracted interest as a potential anticancer compound that modulates different processes in cancer cells (Villota et al., 2021).

In this study, different nanohybrids of AuNPs were biosynthesized with an aqueous extract of *C. arabica* and the anticancer drug doxorubicin (DOX) by direct interaction and by using organic ligands including a short hydrocarbon chain [sodium mercaptopropane sulfonate (MPSNa)] and a long hydrocarbon chain [mercaptoundecanoic acid (MUA)]. These results hold remarkable promise for the use of AuNPs biosynthesized using *C. arabica* extract as specific anticancer drug transporters.

## 2 Materials and methods

Gold standard 0.1% m/m HyCel brand. Green coffee beans from *C. arabica,* Bourbon family, Mundo novo variety, shade grown were obtained from Xicotepec, location: 20°18′49.35″N, 98°0′48.57″W, altitude: 800–1050 m, mean temperature: 18.6°C, mean rainfall: 3181.5 mm, Puebla, Mexico (Ortega-Rivera et al., 2019). Deionised water was obtained from double-distilled water and treated with a Barnstead E-pure three-module deioniser. Nitrocellulose membranes (0.45 μm) were obtained from Millipore. Mercaptoundecanoic acid (C_11_H_22_O_2_S, ≥98%), dimethyl sulfoxide (CH_3_SOCH_3_ ≥ 99.9%), absolute ethanol, sodium hydroxide (NaOH, ≥99.5%), sodium mercapto propane sulfonate (C_3_H_7_O_3_S_2_Na, ≥90%), and MTT [3-(4,5-dimethylthiazol-2-yl) 2,5-diphenyltetrazolium bromide] were purchased from Sigma-Aldrich (Mexico). Dulbecco’s modified Eagle medium, foetal bovine serum, and streptomycin were purchased from Gibco (Life Technologies). Doxorubicin hydrochloride (C_27_H_29_NO_11_ **·**HCl) was obtained from Doxolem^®^ United Kingdom.

### 2.1 Preparing the aqueous extract and phytochemical analysis

An aqueous extract of *C. arabica* was obtained by infusing 3 g of green coffee beans into 20 mL of deionised water for 15 min. The coffee beans were removed, and the obtained extract was filtered through nitrocellulose membranes with a pore size of 0.45 μm. Phytochemical analyses were performed using standard reagents for the determination of metabolites. Standard reagents were used for phytochemical analysis to determine the metabolites, as described below. For **reducing sugars**, Fehling’s test was conducted: 1.0 mL of Fehling’s reagent was added to 1.0 mL of the aqueous extract, homogenised, and placed in a boiling water bath for 5 min. The presence of reducing compounds is considered positive if a brick-red precipitate is formed (Rodríguez-Garza, 2010). Furthermore, Benedict’s test was used: five drops of aqueous extract were added to 2.0 mL of Benedict’s reagent and placed in boiling water for 5 min. A yellow-to-red colour was considered positive (Rodríguez-Garza, 2010). For **carbohydrates,** the Molish test was conducted: three drops of Molish’s reagent were added to2.0 mL of extract, followed by the addition of 1.0 mL of concentrated H_2_SO_4_. The test is positive when a purple ring forms at the interface between the two liquids (Rodríguez-Garza, 2010). For **flavonoids,** the Shinoda test was used: 2 mg of dry extract was diluted in 1.0 mL of ethanol and 0.03 g of Mg filings were added at 60°C, followed by the addition of 3 drops of concentrated HCl. The test is positive if it turns orange, red, pink, blue, or purple (Dominguez, 1973). Cyanidin test: aqueous extract (1.0 mL), hydrochloric alcohol and 0.03 g of Mg filings were added to the aqueous extract. It is considered positive when it turns red or pink (Dominguez, 1973). FeCl_3_ test: 10 drops of 2% aqueous FeCl_3_ were added to 1.0 mL of aqueous extract. It is considered to be positive if it takes on a blue-green colour (Dominguez, 1969). Alkaline medium: three drops of 5% KOH in ethanol were added to 1.0 mL of aqueous extract. It is considered positive if it has a yellow-orange colour (Dominguez, 1969). For **saponins,** a screw-capped test tube was filled with 1.0 mL of the extract and 1.0 mL of deionised water, sealed, and shaken for 3–5 min. It is considered positive if foam is present (Dominguez and Dominguez, 1976). For **tannins,** the FeCl_3_ test was used: three drops of 1% aqueous FeCl_3_ were added to 2.0 mL of aqueous extract, and homogenised. The result is positive if it is a dark green, blue-black, grey-green, yellow, brown, or red precipitate (Domínguez, 1979). Salt gelatin: 10 drops of 0.5% grenetin solution were added to 3.0 mL of aqueous extract. It was considered positive if turbidity and abundant precipitates appeared (Domínguez, 1979). HCl test: 1.0 mL of aqueous extract was placed in a boiling water bath and 0.5 mL of HCl was added and left to react for 10 min. The formation of a precipitate and/or a red colour was considered positive (Rodríguez-Garza, 2010). For **coumarins,** the coumarin test involved adding 2 mL of aqueous extract to a test tube with a lid, placing a strip of filter paper previously soaked in a 6% NaOH solution without touching the extract inside the tube, followed by heating until vapours were formed. A result was considered positive if fluorescent spots were observed under a UV lamp (Robles-García et al., 2016). For antioxidant activity, cation ABTS^·^* was prepared by mixing 7 mM ABTS with 2.45 mM K_2_S_2_O_8_ and diluting using phosphate buffer to an absorbance of 0.7 ± 0.02 at 734 nm. One hundred μL of sample was taken and 1 mL of the ABTS solution was added, incubated for 30 min at 30°C and the absorbance was measured at 734 nm (Fonseca-García et al., 2014).

### 2.2 Biosynthesis and characterization of gold nanoparticles

Gold nanoparticles were synthesised by adding 1 mL of the aqueous extract to 19 mL of 0.13 mM gold solution at 25°C. The pH was adjusted to 7 with sodium hydroxide and incubated at 70°C, with a change in colouration and subsequent confirmation by UV-vis spectroscopy (Spectro UV-VIS Double Beam PC scanning spectrophotometer UVD-2950, Labomed, Inc.) ensuring the formation of gold nanoparticles. The biosynthesized nanoparticles were separated from the reaction medium by centrifugation at 14,000 rpm for 15 min at 25°C, the supernatant was removed, and the precipitate was washed in deionised water three times. The size of the nanoparticles obtained was determined by laser granulometry using the Nanotrac Wave granulometer (Microtrac MRB). Scanning electron microscopy (SEM) (JEOL JSM-6010LA) and transmission electron microscopy (TEM; JEOL JEM-2100) were used to confirm the size and morphology of the particles using Formvar grids (carbon-coated copper). The surface chemistry of the AuNPs was analysed by Fourier-transform infrared spectroscopy–attenuated total reflectance using a Cary 630 FTIR spectroscope (Agilent Technologies) (Ahmed et al., 2016).

### 2.3 Synthesis and characterization of AuNPs-DOX and AuNPs-ligand-DOX hybrids

Two techniques were used for the synthesis of the nanohybrids: direct binding by electrostatic bonding of DOX to the nanoparticles (AuNPs-DOX) and binding of AuNPs to a chemical ligand that was bound to DOX (AuNPs-Ligand-DOX). In the first technique, a solution of AuNPs was mixed with a solution of DOX in a 10:1 M ratio of AuNP:DOX and incubated with stirring for 24 h. Subsequently, the AuNP-DOX nanohybrid was centrifuged, washed, and redispersed in deionised water. In the second technique, two ligands were separately employed: MPSNa and MUA were incubated with the AuNPs for 24 h, centrifuged at 14,000 rpm, and the DOX solution was added at a 10:1 M ratio of AuNPs:DOX (Dhamecha et al., 2016; Zayed et al., 2018). Hybrid stability was analysed using UV-Vis spectroscopy at three pH values, 7.4 (physiological), 6.8 (tumour) and 5.4 (endosomal). For characterisation, the hybrids were analysed by UV-Vis spectroscopy (Spectro UV-VIS Double Beam PC, Scanning Spectrophotometer UVD-2950, Labomed, Inc.) to determine the interaction between the drug and the AuNPs by modifying the characteristic band of the AuNPs using Fourier-transform infrared spectroscopy–attenuated total reflectance (Cary 630 FTIR Agilent Technologies) to detect the presence of the drug on the surface of the AuNPs. Furthermore, SEM (JEOL JSM-6010LA) and TEM (JEOL JEM-2100) was performed to ensure that the morphology and particle size did not undergo significant modifications after the addition of the drug to the surface of the nanoparticles (Coelho et al., 2019).

### 2.4 Cytotoxicity studies of AuNPs, AuNPs-DOX, and AuNPs-ligand-DOX

The anticancer activity of the AuNPs-DOX, AuNPs-MPSNa-DOX, and AuNPs-MUA-DOX nanohybrids was determined in triplicate in cultures of MRC5 non-cancerous cells (from fibroblasts) and H69 cancer cells (from lung cancer) (Salge-Bartels, 2016). They were grown in Dulbecco’s modified Eagle medium supplemented with 10% foetal bovine serum at 37°C in a 5% CO_2_ atmosphere. The cells were incubated with given concentrations of the hybrids, AuNPs, drugs, and ligands. Cell death was determined by the MTT technique involving enzymatic conversion of tetrazolium to formazan (Baneshi et al., 2019). This was quantified using an enzyme-linked immunosorbent assay. Data were analysed using one-way analysis of variance (ANOVA), followed by Tukey’s multiple comparison test using GraphPad Prism 5. In all cases, a value of *p* < 0.05 was considered a significant difference (Dhamecha et al., 2016).

## 3 Results

### 3.1 Biosynthesis and characterization of gold nanoparticles

**FIGURE 1:**
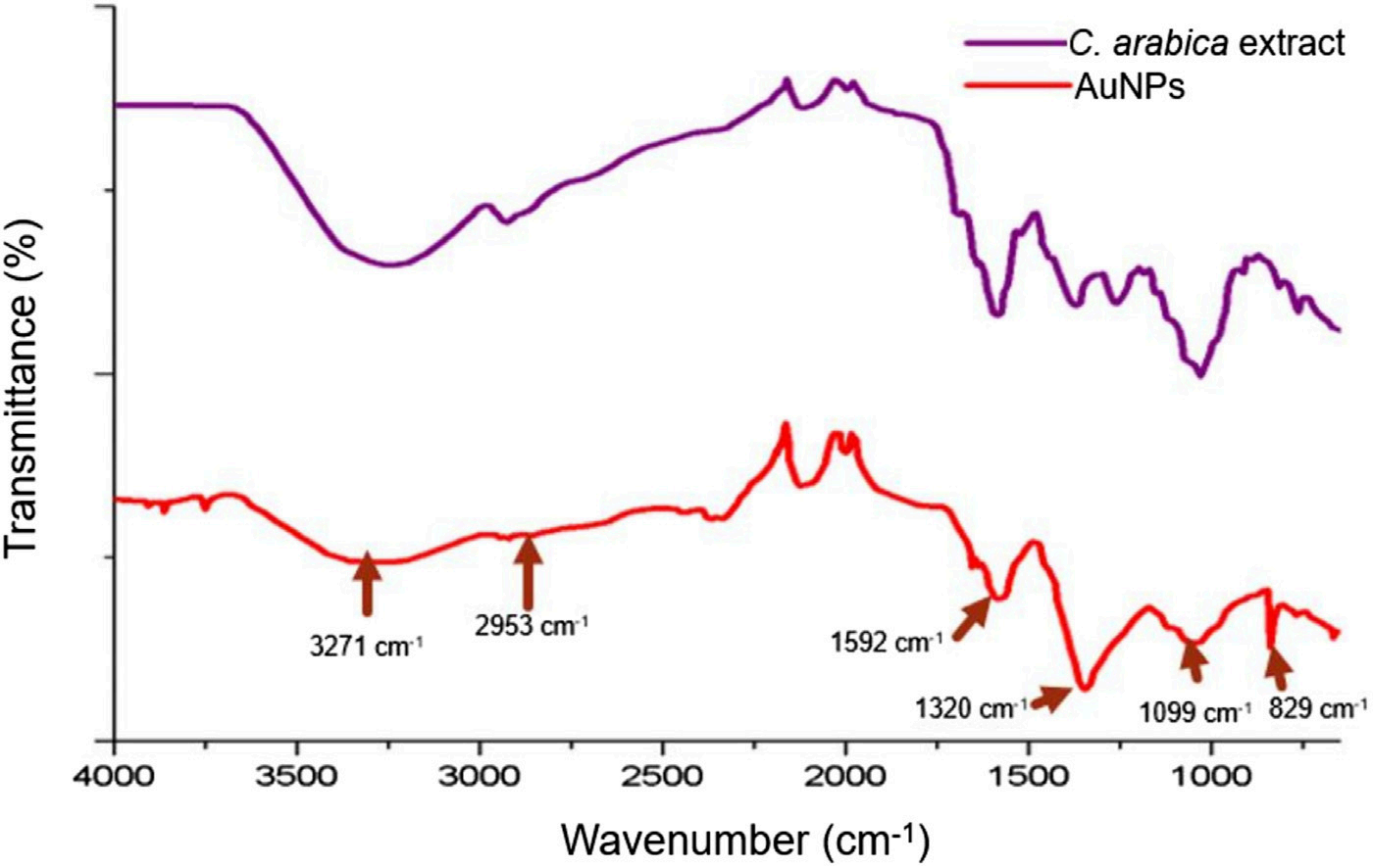
Comparison between the spectra of *Coffea arabica* green bean extract and AuNPs by FT-IR spectroscopy, the functional groups belonging to the phytochemicals that adhere to the surface of the AuNPs are observed (arrows).

The phytochemical determination of the aqueous extract of *C. arabica* green beans confirmed the presence of antioxidant agents, mainly reducing sugars, saponins, flavonoids, tannins, and coumarins. The antioxidant activity of the extract tested by the ABTS^•*^ cation extinction technique produced 94.2% ± 0.6058% activity (Table 1). The antioxidant activity tests of the extract showed an activity of 19.37 ± 0.14 mg/mL TROLOX equivalent. To determine the functional groups of the extract molecules attached to the surface of the nanoparticles, the extracts and nanoparticles were analysed using FT-IR spectroscopy. Characteristic signals of the *C. arabica* extract were observed at 3271 cm^-1^ corresponding to -OH groups at 2953 cm^-1^, characteristic of carbon bonds with sp^3^ hybridisation at 1592 cm^-1^ for C=C (alkene) groups, at 1320 cm^-1^ corresponding to COO^−^ (carboxyl) groups, at 1250 cm^-1^ belonging to C-O-C (ether) groups, at 1099 cm^-1^ corresponding to cyclic and aliphatic compounds, and finally a signal at 829 cm^-1^ characteristic of aromatic compounds (Figure 1). Meanwhile, the signals of the -OH groups of alcohols and phenols located around 3300 cm^-1^ were preserved on the surface of the nanoparticles, as well as aliphatic carbons located at 2953 cm^-1^. The signal at 1592 cm^-1^ corresponds to C=C alkenes, while that at 1320 cm^-1^ reflects -OH bonds of phenols and carboxylic acids COO^−^, a non-conserved peak is located at 1250 cm^-1^ that corresponded to the C-O-C group of aliphatic compounds, a signal was also observed at 1099 cm^-1^ that is characteristic of C-O-C ethers, but in cyclic compounds; and at 829 cm^-1^ corresponded to aromatic compounds (Lambert et al., 1998; Lambert et al., 2010).

**TABLE 1:** Main phytochemical compounds present in the green bean extract of *Coffea arabica*. Antioxidant activity of the extract by ABTS^·^* cation.

Metabolite/test	Result
Reducing sugars	Positive
Saponins	Positive
Flavonoids	Positive
Tannins	Positive
Coumarins	Positive
ABTS^·^* cation TROLOX equivalent	94.2% ± 0.6058% 19.37 ± 0.14 mg/mL

**FIGURE 2:**
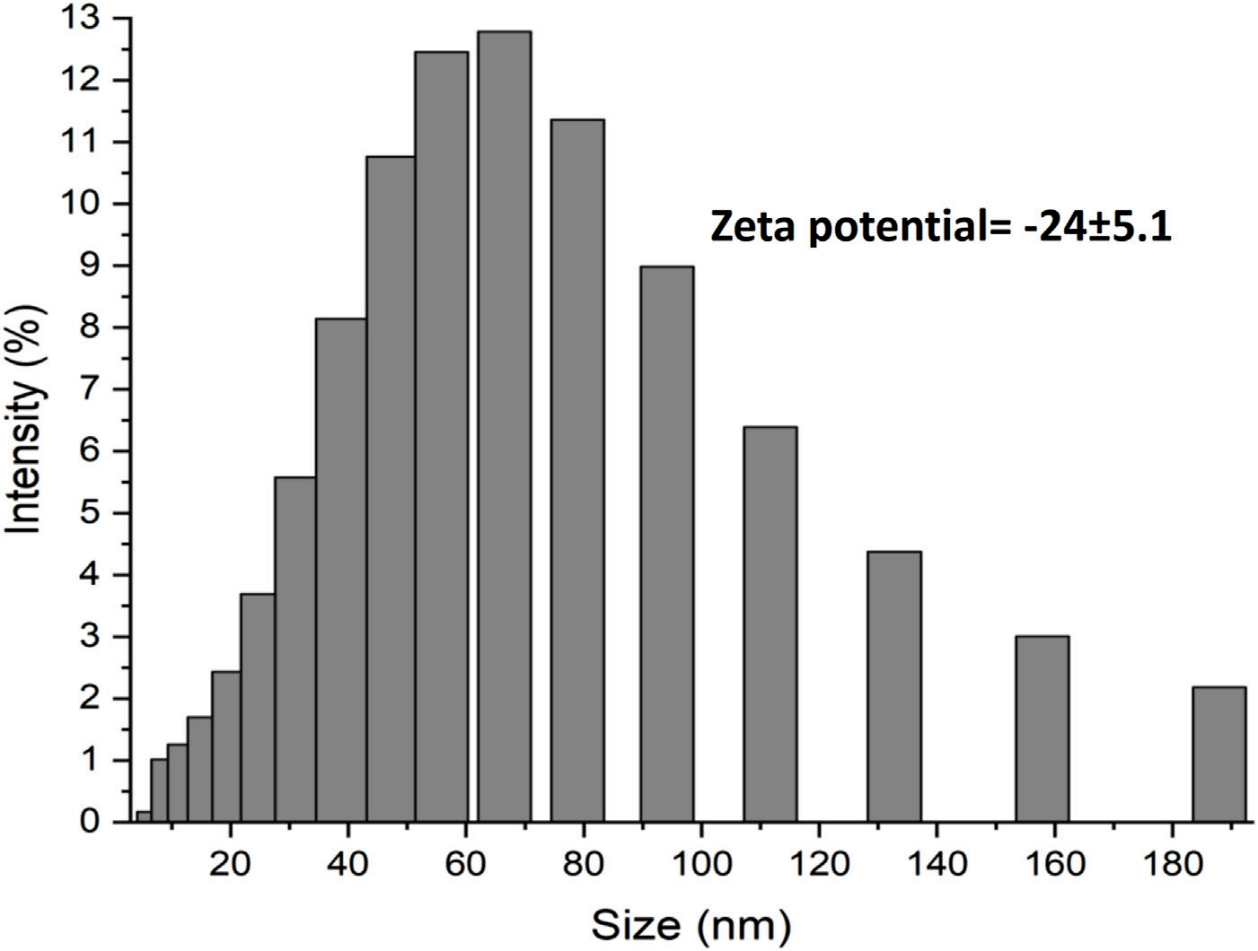
Analysis of the size distribution by laser granulometry and determination of the Zeta potential, two of the most important properties, limiting in the application of AuNPs.

**FIGURE 3:**
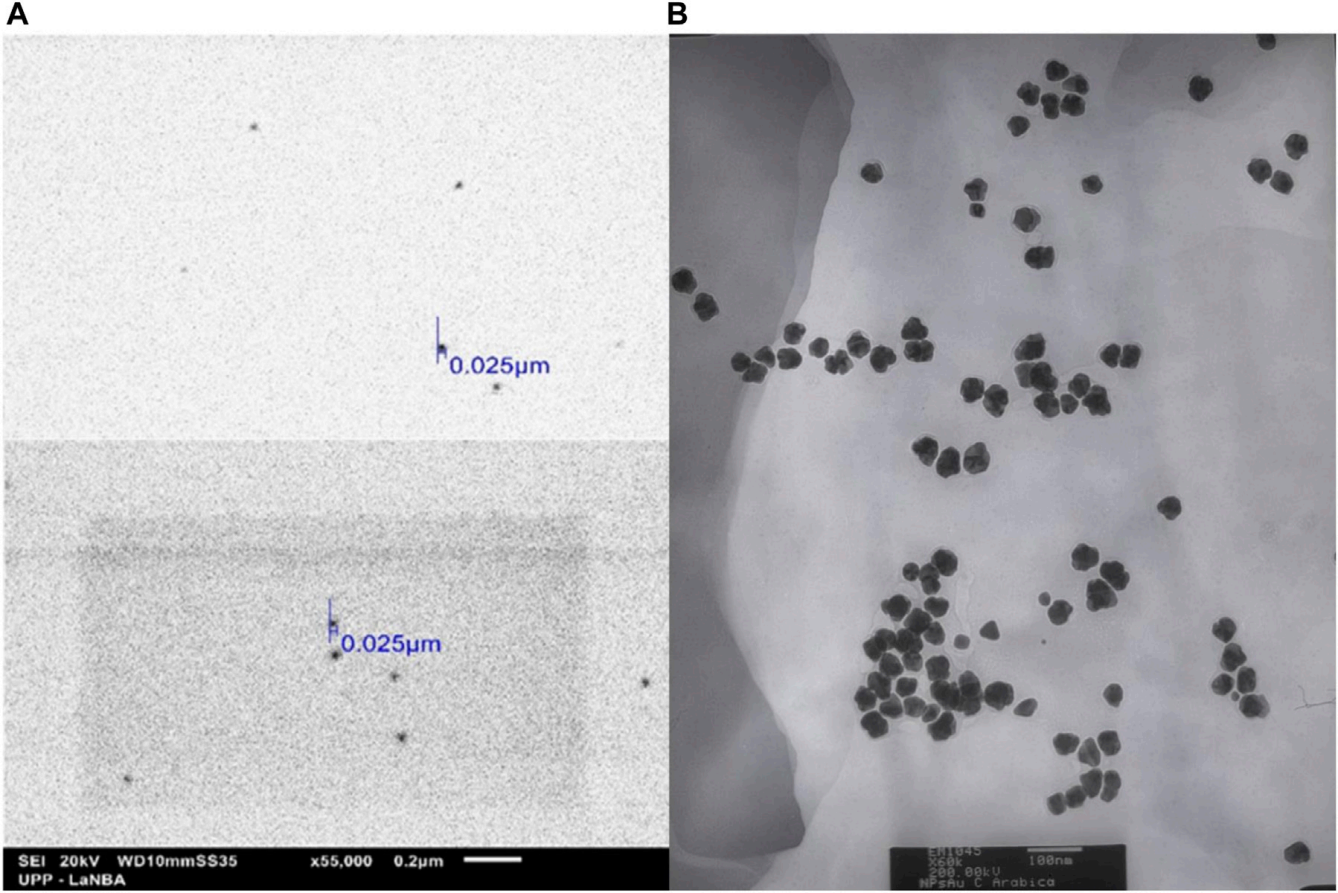
Micrographs obtained by **(A)** SEM (×55,000 magnification) and **(B)** TEM (×60,000 magnification), of the AuNPs biosynthesized with *Coffea arabica*, although they are not completely spherical, they are monodispersing and of a homogeneous morphology, uncommon in biosynthesis methods.

Ultraviolet-vis spectroscopy was performed to confirm the formation of AuNPs; a characteristic band was obtained with a peak at 517 nm, which is within the reported range (Kumari et al., 2019). The particle size analysis showed a distribution with an interval from 8.61 to 187.83 nm, with a mode at 66.29 nm. Eighty-five percent of the nanoparticles were located below 100 nm, with a Z potential of −24.0 ± 5.0 mV (Figure 2). Figure 3A shows the size of the 25 nm AuNPs and their spherical morphology by SEM. However, the AuNPs presented irregularities in their morphology by TEM (Figure 3B), since a spherical particle projects a perfect circle in a two-dimensional image. Analysis by ImageJ^®^ software determined the average circularity index of 0.871 ± 0.0393, where an index of 1 is equivalent to a circle and therefore to a sphere. Similarly, ImageJ^®^ determined an average diameter of 41.701 ± 6.911 nm, which was far from that obtained by laser granulometry which was 66.97 nm as mentioned by Keijok et al. (2019). The diameter obtained by TEM would be the closest to reality since laser granulometry has the tendency to show larger diameters if two or more particles are very close to each other.

### 3.2 Synthesis and characterization of the AuNPs-DOX and AuNPs-ligand-DOX hybrids

**FIGURE 4:**
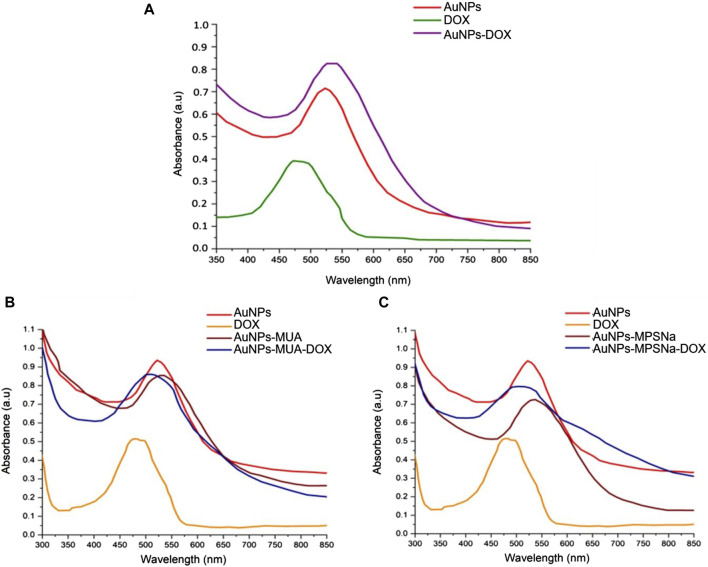
UV-vis spectra of the hybrids: **(A)** AuNPs-DOX, **(B)** AuNPs-MUA-DOX, **(C)** AuNPs-MPSNa-DOX. The closeness between the band of AuNPs and DOX causes a broadening of the band in the hybrids and denotes the presence of the drug on their surface (red AuNPs, orange DOX, brown AuNPs-ligand, blue AuNPs-ligand-DOX.

**FIGURE 5:**
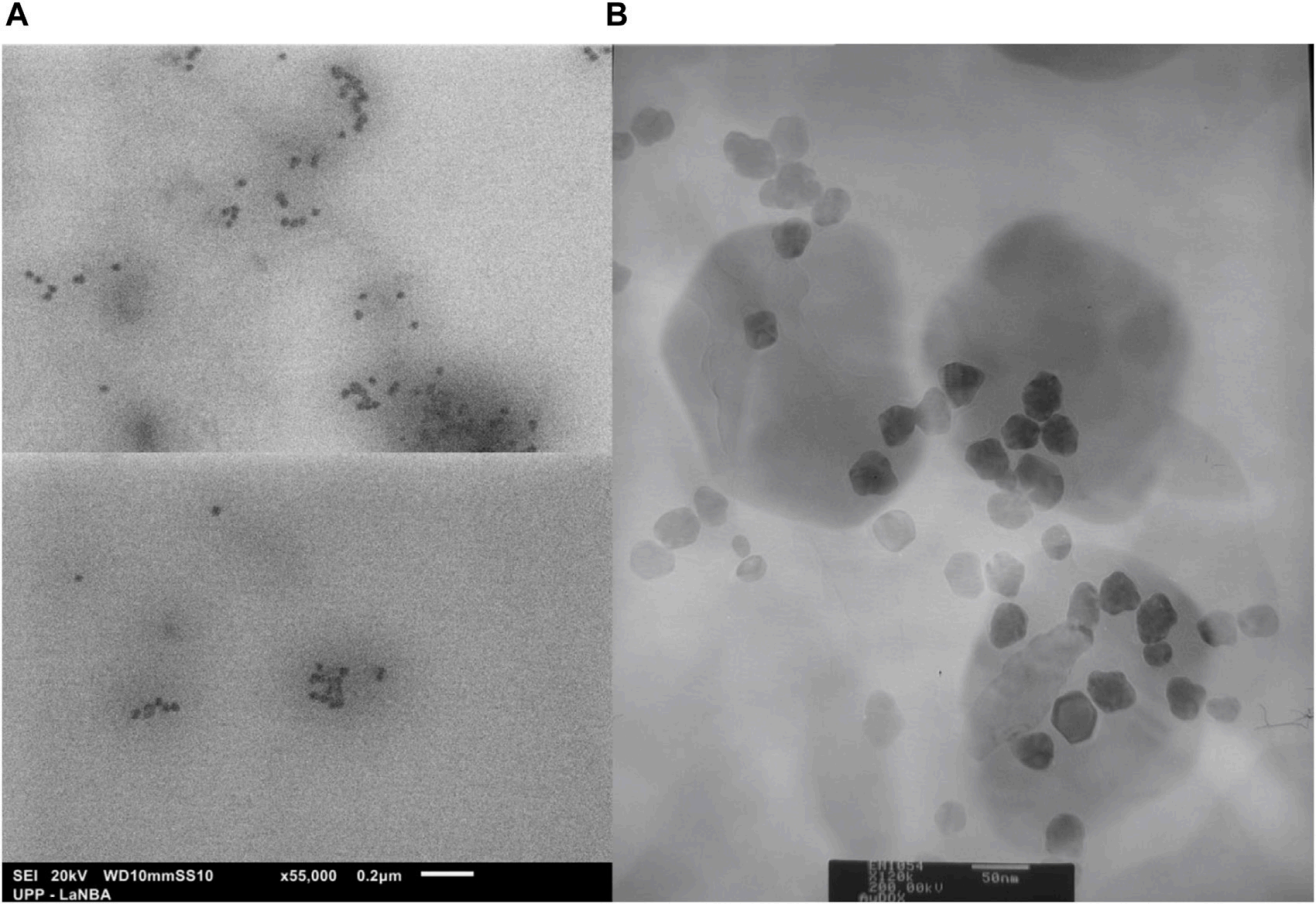
Morphology and size of nanoparticles of the AuNPs-DOX hybrid: **(A)** SEM (×55,000 magnification) and **(B)** TEM (x120,000 magnification). Organic matter is observed surrounding the nanoparticles, presumably DOX and phytochemicals.

**FIGURE 6:**
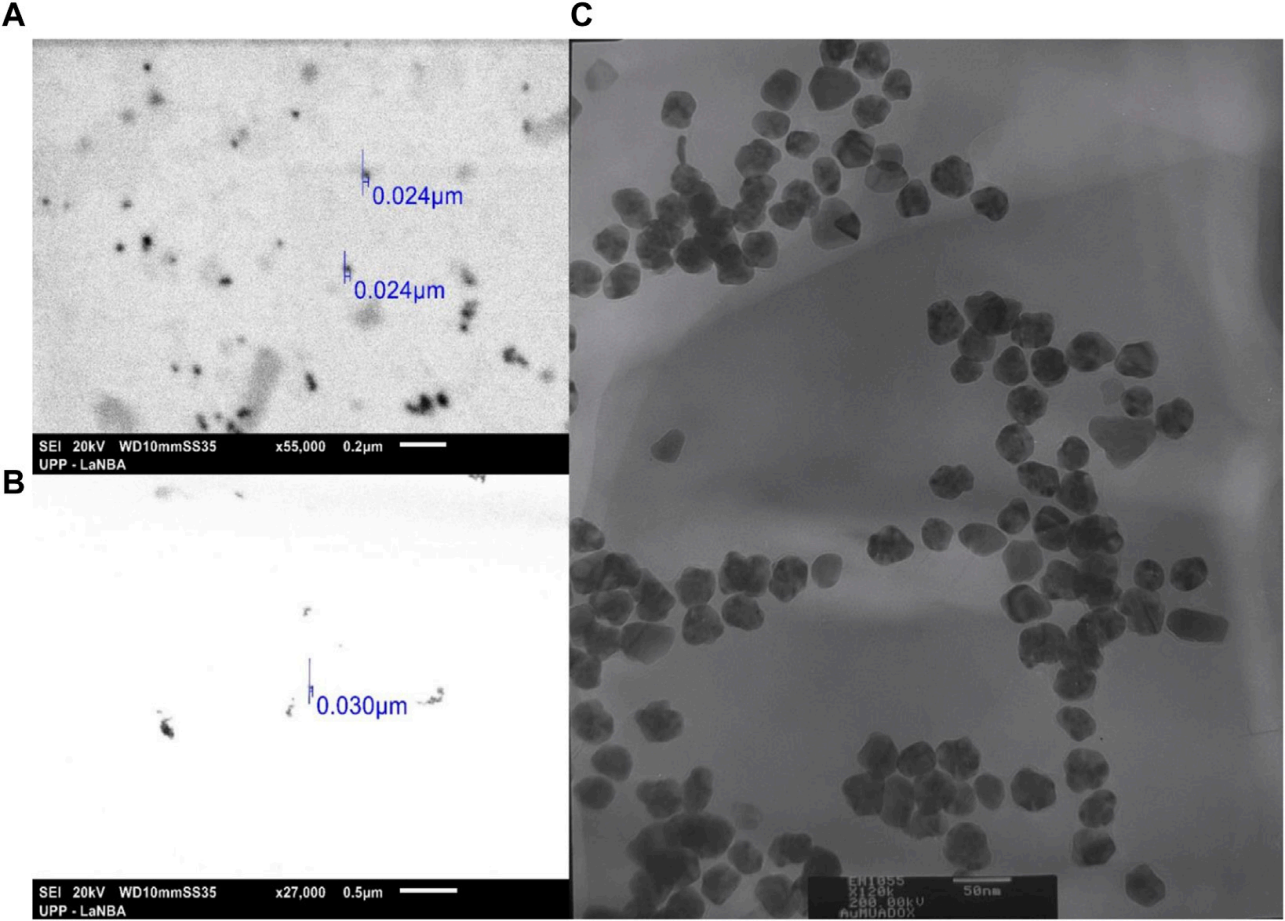
Morphology and size of the hybrids with the ligand: **(A)** AuNPs-MUA-DOX by SEM (×55,000 magnification), **(B)** AuNPs-MPSNa-DOX by SEM (×27,000 magnification) and **(C)** AuNPs-MUA-DOX by TEM (x120,000 magnification). Despite adding more organic charge to the surface of the nanoparticles, the hybrids maintain the repulsion between them, showing individual nanoparticles without agglomerations.

Doxorubicin has the property of protonating in an aqueous medium so it can form electrostatic bonds with the AuNPs (negatively charged based on the determination of the Z potential), which allows the bonding between them by electrostatic forces to form the AuNP-DOX hybrid. This was confirmed by UV-vis spectroscopy (Figure 4) since the addition of DOX (Figure 4A) caused a hyperchromic and bathochromic effect with respect to the characteristic band of the AuNPs, indicative of modifications on the surface of these nanoparticles (Teng et al., 2017). Similarly, the binding of the MUA ligand caused hyperchromic and bathochromic effects (Figure 4B), unlike the MPSNa ligand, whose effect was only bathochromic (Figure 4C). Finally, the addition of DOX caused a hypochromic and hypsochromic effect in the hybrid, which was similar to that of DOX in solution (Figures 4B, C) using the property of covalent binding of gold to sulphur (Nuzzo and Allara, 1983). Using a ligand with a negative functional group, binding by electrostatic interactions is also favoured, wherein the ligand is covalently bound to the nanoparticle by a thiol group (Tortiglione and de la Fuente, 2019). The size of the hybrid was determined by SEM and TEM, with an approximate size of 37.05 ± 7.32 nm in the case of AuNPs-DOX (Figure 5), as well as 30 nm for AuNPs-MPSNa-DOX and 35.32 ± 6.34 nm for AuNPs-MUA-DOX (Figure 6). All sizes were within the range of 20–40 nm (Figures 5, 6).

**FIGURE 7:**
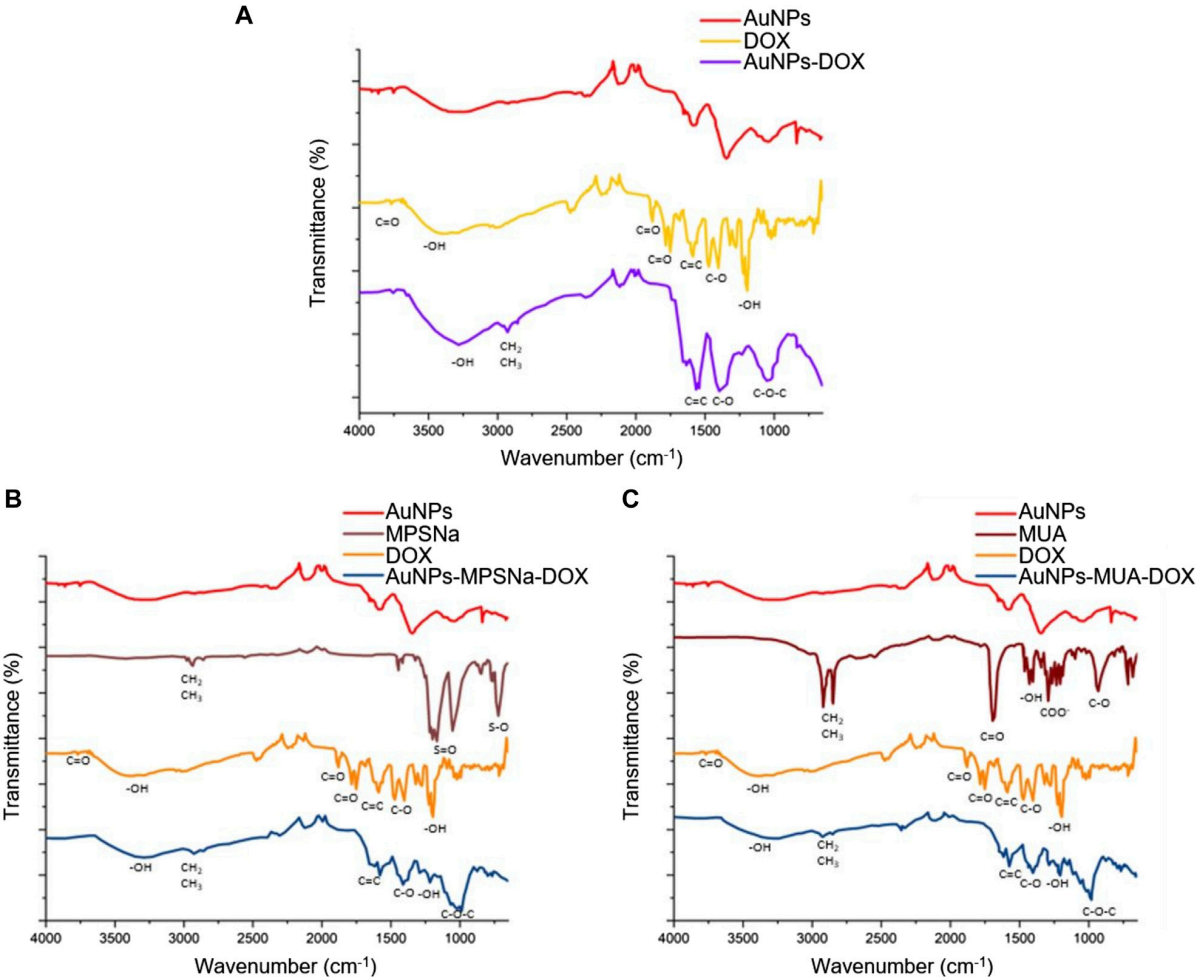
Comparison of the spectra of the obtained hybrids and their components by FT-IR spectroscopy: **(A)** AuNPs-DOX; **(B)** AuNPs-MPSNa-DOX and **(C)** AuNPs-MUA-DOX. The characteristic functional groups of each compound as well as in the hybrid are shown, confirming the ligand and drug loading.

The analysis of the spectral signals of the hybrids by FT-IR allowed us to determine that the characteristic functional groups of the ligand and the drug in the two synthesis routes were conserved in the hybrids and on the surface of the nanoparticle (Figure 7) in the AuNP-DOX hybrid (Figure 7A). The characteristic signal for -OH groups is observed in the hybrids at 3260 cm^-1^, while the signal corresponding to C=C double bonds of cyclic compounds of the DOX structure occurs at 1615 cm^-1^, the signal belonging to the C-O group occurs at 1360 cm^-1^ and a signal of the C-O-C ether group present in sugars occurs at 1090 cm^-1^. Meanwhile, both AuNPs-MPSNa-DOX and AuNPs-MUA-DOX present the same signals (Figures 7B, C). However, these signals showed unfolding, attributable to interactions with the ligand (Figure 7B) since they belonged to negatively charged groups (Lambert et al., 1998).

**FIGURE 8:**
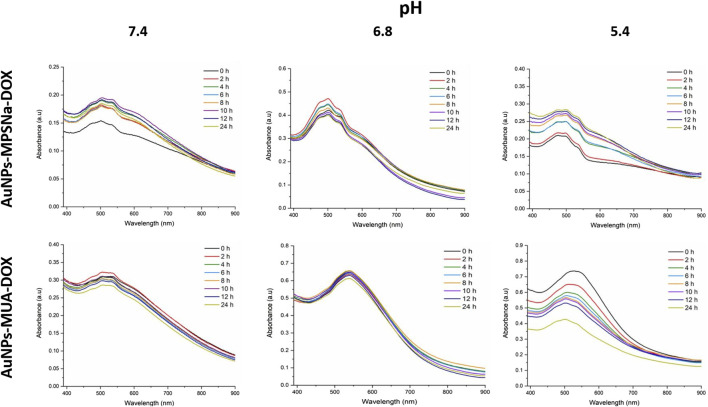
Kinetics obtained by UV-vis of the hybrids AuNPs-MPSNa-DOX (above) and AuNPs-MUA-DOX (below), at pH 7.4, pH 6.8 and pH 5.4, the characteristic bands are modified depending on the pH used, changing the absorption maximum and moving towards lower wavelengths where the maximum absorption of DOX is found, as well as the amplitude of this, evidencing the separation of the components of the hybrids.

The colloidal stability of these hybrids were determined by placing them at different pH values. The stability of the hybrid depends on the ligand since the hybrid agglomerates at pH 7.4 when using the MPSNa ligand (Figure 8). The same occurred at pH 6.8, which is reflected in a decrease in the absorbance of the characteristic band, unlike the MUA ligand (Figure 8) whose absorbance remained constant at pH 7.4 and pH 6.8. Meanwhile, there was instability and agglomeration of the hybrids with both ligands at a more acidic pH (5.4) (Figure 8) because the absorbance of the characteristic band of the AuNPs (520 nm) decreased owing to the proximity of the DOX signal (480 nm). A decrease in the absorbance of the AuNPs and an increase in the absorbance of the drug were observed at pH 5.4, while the signal of the drug was almost exclusively observed at 24 h.

### 3.3 *In vitro* cytotoxicity analysis on H69 (cancerous) and MRC5 (non-cancerous) lung cells

**FIGURE 9:**
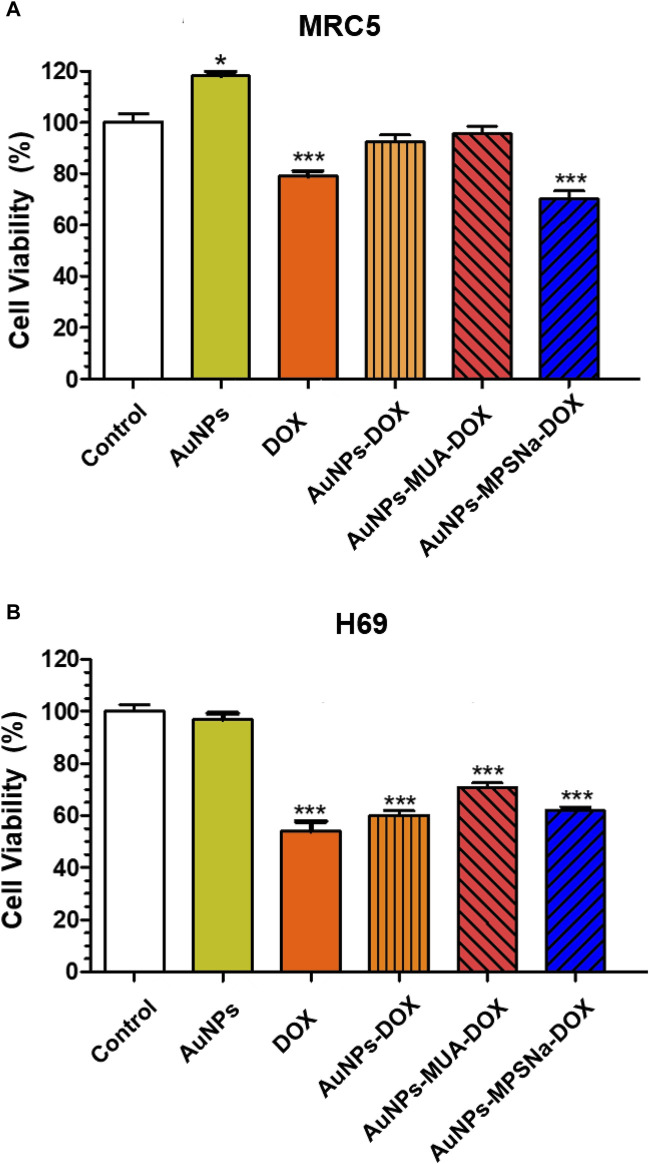
Effect of the hybrids AuNPs-DOX on cell viability. **(A)** MRC5 cells (non-cancerous) incubated 24 h with the different hybrids and controls. **(B)** H69 (cancerous) cells incubated 24 h with the different hybrids and controls. *p ≤ 0.05, ***p ≤ 0.0001, one-way ANOVA post-test Tukey HSD.

As expected, DOX showed cytotoxicity for H69 cancerous and MRC5 non-cancerous cell types since cell viability significantly decreased (*p* ≤ 0.0001 one-way ANOVA, Tukey post-test) (Figure 9A). Interestingly, the AuNPs prevented DOX-induced damage in healthy cells, since neither the AuNP-DOX hybrid nor AuNP-MUA-DOX had a negative effect on cell viability (Figure 9A). However, cell viability significantly decreased when the AuNPs-MPSNa-DOX hybrid was used (*p* < 0.0001, one-way ANOVA, Tukey’s post-test). In H69 cancer cells, both DOX and the AuNPs-DOX, AuNPs-MUA-DOX, and AuNPs-MPSNa-DOX hybrids had cytotoxic effects (Figure 9B) (*p* < 0.0001 for all groups, one-way ANOVA, post-test Tukey). Notably, AuNPs alone had no negative effects on healthy or cancerous cells.

## 4 Discussion

### 4.1 Biosynthesis and characterization of gold nanoparticles

Coffee is one of the most important beverages worldwide. There were 10.3 million tons of coffee produced in 2022, as indicated by the World Coffee Organization. Obtaining the extract from roasted beans or spent beans (the main form of utilization of coffee) results in the loss or transformation of many phytochemicals (chlorogenic acids, and phenolic compounds such as flavonoids and terpenes) (Priftis et al., 2018) since they are not thermostable during roasting. The unmodified phytochemicals provide a favourable anticarcinogenic effect (Yust et al., 2022). Therefore, it was decided to use green coffee beans to obtain the extract, in which we could detect the presence of such compounds (in addition to reducing sugars). This allowed the formation and stabilization of monodisperse gold nanoparticles in a single step resulting in an average diameter of 41.701 ± 6.911 nm (Keijok et al., 2019), as observed in Figure 3. The preferential size for application in cancer treatment is approximately 50 nm (a size between 20 and 40 nm is less cytotoxic towards healthy cells) (Yang-Peng et al., 2017). Similarly, the Z potential of −24.0 ± 5.0 mV was favourable owing to the properties of DOX to protonate in aqueous medium since extra steps would have been required in the methodology to allow drug loading, in addition to obtaining these AuNPs to allow the replacement of those obtained by the citrate method (Z potential of −29.18 mV) if a positive Z potential was obtained as in the case of Keijok et al. (2019). Doxorubicin and other commonly used anticancer drugs do not bind to other types of metal nanoparticles such as TiO_2_ (Garcia-Contreras et al., 2014). Therefore, the use of coffee extract in the biosynthesis of AuNPs improves drug interactions and has an anticancer effect.

### 4.2 Synthesis and characterization of AuNPs-DOX and AuNPs-ligand-DOX hybrids

One of the main objectives of adding any molecule or compound to the surface of nanoparticles is to improve their properties, such as solubility in physiological media, stability, loading, and unloading of the drug into the cells of the target area, thus seeking good cellular uptake, which is the result of the size, morphology, and surface charge (Darweesh et al., 2019). Thus, obtaining hybrids with particle sizes within 20–40 nm ensures specificity towards tumour cells and decreases the chances of cytotoxicity towards healthy cells (Yang-Peng et al., 2017; Darweesh et al., 2019).

Regarding the decrease in absorbance in the UV-vis range of the hybrid characteristic band, it is interpreted as a clumping of the hybrids (Zayed et al., 2018) and drug release (Khutale and Casey, 2017), similar to what is reported by other authors (Dhamecha et al., 2016; Ravichandran et al., 2016). The decrease in pH causes agglomeration and drug release which is especially observed in hybrids possessing DOX owing to the shape and closeness between the characteristic bands of the hybrids and DOX. However, a protective effect is exerted by the MUA ligand since it prevented unwanted agglomeration and release of DOX, as if it were a high molecular weight polymer, since it had the same effect as the one synthesized by Zayed et al. (2018).

### 4.3 *In vitro* cytotoxicity analysis on H69 (cancerous) and MRC5 (non-cancerous) lung cells

Doxorubicin inhibits DNA and RNA synthesis by intercalating DNA base pairs and inhibiting topoisomerase II and steric hindrance. The anthraquinone fragment present in anthracyclines is responsible for cytotoxic damage to cells through the generation of reactive oxygen species and subsequent induction of the apoptotic pathway (Siveski-Iliskovic et al., 1995). Reactive oxygen species generation was first documented in 1975 and DOX generates free radicals through an oxidoreduction cycle (Goodman and Hochstein, 1977). Therefore, this drug is considered a highly selective generator of reactive oxygen species (Avendaño and Menéndez, 2015). In this study, the *in vitro* assays stand out from other similar reports; for example, Zayed et al. (2018) tested AuNPs-DOX conjugates linked by electrostatic interactions, although unlike the present one, the nanoparticles were obtained by citrate reduction; they obtained a reduction in MCF-7 cell viability of 15.4% at 10 ppm, while in our case with H69 cells, it was reduced up to 40% at 50 ppm; however, Zayed et al. did not analyse cytotoxicity towards healthy cells. Similarly, Khutale and Casey in 2017 analysed Au-PEG-PAMAMAM-DOX hybrids on A549 cells and obtained a reduction in the percentage of viability of up to 70%; therefore, it is likely that the hybrids present a greater reduction in cell viability on other cell lines, as in the case of Du et al. (2017) wherein the IC_50_ of DOX was reduced from 26 ± 3 to 10 ± 4 nM, employing A2780 cells. A further example of the above was obtained by Madhusudhan et al. (2020), who reported that using 10 ppm of their conjugate reduced cell viability by up to 75% in LN-229 cells. Therefore, the type of cells used in the analysis is an important factor to consider.

## 5 Conclusion

In conclusion, the combination of monodisperse AuNPs obtained by green synthesis using *C. arabica* extract, doxorubicin, and commercial organic ligands (MUA) was highly effective in increasing specificity and cytotoxicity in H69 tumour cells. This innovative strategy offers a promising approach for cancer treatment, taking advantage of the unique properties of each component to improve therapeutic efficacy and reduce unwanted side effects (Vázquez-Vivar et al., 1997; Thambiraj et al., 2018; Maeda, 2021).

## Data Availability

The original contributions presented in the study are included in the article/supplementary material, further inquiries can be directed to the corresponding author.

## Abbreviations

ANOVA, analysis of variance; AuNPs, gold nanoparticles; DOX, doxorubicin; MPSNa, sodium mercaptopropane sulfonate; MUA, mercaptoundecanoic acid; SEM, scanning electron microscopy; TEM, transmission electron microscopy.
